# SOX8 Affects Tumoral SPARC Expression by Regulating EZH2 to Attenuate Effectiveness of albumin-bound paclitaxel in PDAC

**DOI:** 10.7150/ijbs.64752

**Published:** 2022-01-01

**Authors:** Shuai Yuan, Jie Xu, Bodong Zhou, Yizhang Zhou, Mingxiao Lang, Junli Cao, Zhe Liu, Shengyu Yang, Song Gao, Jihui Hao

**Affiliations:** 1 Department of Pancreatic Cancer, Tianjin Medical University Cancer Institute and Hospital, National Clinical Research Center for Cancer, Key Laboratory of Cancer Prevention and Therapy, Tianjin, China.; 2 Senior Ward, Tianjin Medical University Cancer Institute and Hospital, National Clinical Research Center for Cancer, Key Laboratory of Cancer Prevention and Therapy, Tianjin, China.; 3 Department of Lymphoma, Tianjin Medical University Cancer Institute and Hospital, National Clinical Research Center for Cancer, Key Laboratory of Cancer, Prevention and Therapy, Tianjin, China.; 4 Department of oncology, First Hospital of Qinhuangdao, Qinhuangdao, China.; 5 Department of Immunology, Tianjin Medical University, Tianjin, China.; 6 Department of Cellular & Molecular Physiology, Penn State College of Medicine, Hersy, U.S.

**Keywords:** PDAC, albumin-bound paclitaxel, SOX8, chemo-resistance, EZH2, SPARC

## Abstract

Pancreatic cancer is a dismal malignancy with poor prognosis. In spite of progress in surgical technology, chemotherapy is still the cornerstone in the multi-disciplinary treatment. Albumin-bound paclitaxel is a first-line treatment for PDAC patients. Yet the response rate of the drug is far from satisfying. SOX8 is a member of the sex determining region Y-boxes family, which is potentially related to the chemoresistance of tumor. Patient with high expression of SOX8 were insensitive to albumin-bound paclitaxel. SOX8 reduced apoptosis and G2/M cell cycle arrest caused by albumin-bound paclitaxel. SOX8 transcriptionally regulated EZH2, which reduced expression of SPARC by promoting the methylation of SPARC, thereby reducing the transport of albumin-bound paclitaxel in pancreatic cancer cells. EZH2 inhibitor, UNC1999, can reverse the effect of SOX8 on chemo-resistance of albumin-bound paclitaxel. Collectively, our data revealed SOX8/EZH2/SPARC signaling induced primary chemo-resistance of albumin-bound paclitaxel in pancreatic ductal adenocarcinoma.

## Introduction

Pancreatic ductal adenocarcinoma (PDAC) remains a highly fatal malignancy and is projected to become the second leading cause of cancer-related death in the next twenty to thirty years. The 5-year survival rate at the time of diagnosis is 10% in the USA 1. Over the past decade, improvements in surgical treatment for PDAC have made indistinctive progress in patient prognosis. Systemic chemotherapy is still a potent option in comprehensive therapy 2. Gemcitabine had been the standard regimen of PDAC for many years until recently, when the combination of albumin-bound paclitaxel (Nab-p) with gemcitabine was found to significantly improve patients' outcomes in a large, randomized trial 3. The addition of albumin-bound paclitaxel to gemcitabine may prolong the overall survival of PDAC patient by nearly two months, yet only approximately 33% patients' exhibit response to the novel regimen 4. Moreover, there is a lack of biomarkers predicting therapy response. Stromal secreted protein acidic and rich in cysteine (SPARC) was once considered a potential predictive biomarker. However, further studies reported controversial conclusions 5. Given the role of SPARC in transporting albumin-bound paclitaxel into tumor cells 6, tumoral SPARC may be a more straightforward biomarker for predicting the effectiveness. Therefore, it is meaningful to reveal the mechanism of tumoral SPARC regulation in PDAC cell to improve the efficiency of albumin-bound paclitaxel.

Sex-determining region Y-boxes (SOX) family members are expressed in multiple types of stem and progenitor cell types and play a pivotal role in the regulatory network of embryonic development 7. In the SOX family of transcription factors, the SOXE group has three members, including SOX8, SOX9 and SOX10 8. It has been known that SOX8 acts as a mediator to promote chemoresistance in triple-negative breast cancer 9. SOX8 also interacts with other co-factors to confer chemoresistance and stemness properties in chemo-resistant TSCC 10. Considering the oncogenic role of SOX8 in chemoresistance, it is of great importance to clarify the functions and clinical significance of SOX8 in PDAC.

In this study, we explored the functions and molecular mechanisms of SOX8 in albumin-bound paclitaxel primary chemoresistance in PDAC. Our data demonstrated that PDAC patients have higher SOX8 expression than healthy people. The differential expression of SOX8 was closely correlated with nab-paclitaxel efficacy. Mechanistically, SOX8 promotes enhancer of zeste homolog 2(EZH2) transcription by binding to the EZH2 promoter. Subsequently, EZH2 suppressed tumoral SPARC expression through methylation. Functionally, upregulation of SOX8 in PDAC cells attenuates the efficacy of albumin-bound paclitaxel *in vivo* and *in vitro*. Inhibition of EZH2 may reverse the above effect. Collectively, our data uncovered SOX8/EZH2/SPARC pathway drives albumin-bound paclitaxel resistance in PDAC.

## Material and Methods

### Ethics statement

Human tissue specimens collected for this study required written informed consent from the donor (Ek2020037). The animal procedures (NSFC-AE-2020024) were conducted in accordance with the standards set forth in the Guide for the Care and Use of Laboratory Animals [eighth edition, National Institutes of Health (NIH)].

### Cell cultures

Human pancreatic cancer cell lines Panc-1 and CFPAC-1 were obtained from the Committee of Type Culture Collection of Chinese Academy of Sciences (Shanghai, China). Panc-1 cells were cultured in Dulbecco's modified Eagle medium (DMEM) (Thermo Fisher Scientific, USA) medium with 10% fetal bovine serum and 1% penicillin-streptomycin solution (100 U/ml) in the incubator containing 5% CO2 at 37 °C. CFPAC-1 cells were cultured in IMDM (Thermo Fisher Scientific, USA) medium with 10% fetal bovine serum and 1% penicillin-streptomycin solution (100 U/ml) in the incubator containing 5% CO2 at 37 °C. Panc-1 SOX8 expressing cells were also cultured in DMEM and CFPAC-1 SOX8 knock-down cells were also cultured in IMDM.

### Western blot analysis

PDAC cells or tumor tissue homogenate were solubilized in SDS lysis with 1% phosphatase inhibitor and 1% SDS lysis buffer cocktail on ice. Use the BCA protein assay kit (Pierce) to determine the protein concentration. Protein was mixed with 5× loading buffer and boiled at 100 °C for 10 minutes. The protein (20 μg/lane) was loaded into the lane of an SDS-polyacrylamide gel and separated by electrophoresis and then transferred to the PVDF membrane at 260 mA for 120 minutes. Membranes were soaked with antibodies. Enhanced chemiluminescence (Pierce) was used to detect specific proteins. Information of antibodies used in this study was shown in Table S1.

### Transient transfection with siRNAs

The siRNAs target EZH2 (siEZH2) and SPARC (siSPARC) were purchased from Gene Pharma. The siRNAs (10 nM) were transfected to PDAC cells with Lipofectamine 2000 transfection reagent (Invitrogen) follow the manufacturer's instructions.

### RT-PCR analysis and real time-PCR

Total RNA of PDAC cells were isolated with TRIzol Reagent (Invitrogen) and the First-Strand Synthesis System (Takara) was used for first-strand cDNA synthesis. Each sample was processed in triplicate and each experiment was repeated at least three times independently. β-actin was used as loading control. The cDNA was used as template for q-PCR. 2x SYBR Green qPCR Master Mix (Bimake) and CFX96 Touch™ Real-Time PCR Detection System (Bio-Rad) were used in the q-PCR experiment. Primer sequences used in this study were shown in Table S2.

### Flow cytometry

Cell cycle and apoptosis of cells were detected by BD FACS Canto II. Apoptosis was detected with APC Annexin V Apoptosis Detection Kit (eBioscience) with 7-AAD according to Manufacturer's instructions. Cell cycle of cells was detected with Cell cycle and apoptosis detection kit (Beyotime).

### Immunohistochemistry (IHC)

PDAC tissues and subcutaneous tumors were fixed with Formalin and embedded with paraffin, and then slices into 5 μm sections. The sections were boiled in EDTANa2 buffer to retrieval the antigen and were incubated with anti-SOX8 (1:100, rabbit, polyclonal, catalog no. ab104245, abcam, USA), anti-EZH2 (1:400, rabbit, monoclonal, catalog no. 5246, CST, USA), anti-SPARC (1:100, rabbit, monoclonal, catalog no. ab207327, abcam, USA) overnight at 4 °C. DAB Kit (maxim) was used for color development.

### Methylation specific PCR (MSP)

DNA was isolated from the cell lines using DNAzol reagent (Invitrogen Life Technologies). The process of bisulfite conversion of the DNA samples and the method to determine methylation status of the SPARC gene were derived from a published study 11.

### Chromatin immunoprecipitation assay (ChIP)

Chromatin immunoprecipitation assay was performed using a commercial kit (EZ-ChIP 17-371, Merk) according to the manufacturer's instructions.

### Animal studies

The 4-week-old female nude mice were placed in a barrier facility on the HEPA filter rack. All animal studies were conducted in accordance with the principles and procedures outlined in the NIH guidelines for the care and use of laboratory animals and under approved protocols. Albumin bound paclitaxel was obtained from Institute of oncology and hospital of Tianjin Medical University. Cells were obtained by trypsin digestion, washed with PBS and resuspended in Matrigel diluted with PBS in the ratio 1:1 at the rate of 1×10^7^ cells/ml, and then subcutaneously injected into both sides of mice (1×10^6^ cells). Seven days after xenotransplantation, 15 nude mice were randomly divided into 4 groups (n = 5 each): normal saline group, UNC1999 group, nab-p group, nab-p plus UNC1999 group). The volume of primary tumor was calculated as ABC × 0.5. After 35 days, the primary tumor was removed from the lateral abdomen of mice. The tumors were fixed in formalin and embedded in paraffin for IHC analysis.

### Statistical analysis

The data are presented as “mean ± SE” and analyzed by SPSS 19.0. The differences between two groups were assessed by Student's t-test. P< 0.05 is considered statistically significant.

## Results

### Expression of SOX8 is increased in PDAC tissues and PDAC with high SOX8 expression is insensitive to nano albumin-bound paclitaxel

**Figure 1:**
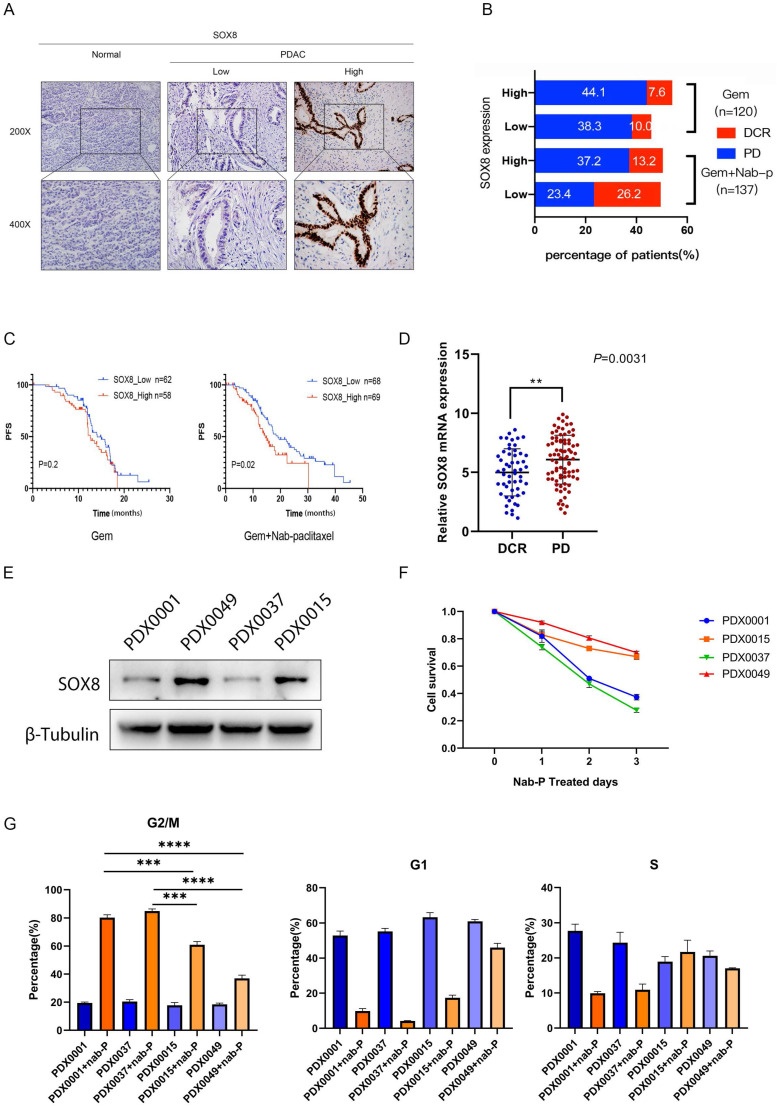
** SOX8 elevated expression in PDACs indicates insensitivity to nab-P. (A)** Expression of SOX8 in PDACs. **(B)** Different expression of SOX8 in DCR/PD patients. **(C)** Association of SOX8 expression with PFS rate in PDAC patients received treatment of GEM/GEM+nab-P. **(D)** mRNA expression of SOX8 in DCR/PD patients. **(E)** Western blot analysis of SOX8 expression in primary PDAC cells. **(F)** Cell survival of primary PDAC cells treated with nab-P and G. cell cycle.

In order to verify the expression of SOX8 in pancreatic cancer, we collected postoperative specimens of 257 PDAC patients and performed IHC staining. The results showed that the expression of SOX8 in pancreatic cancer was significantly higher than that of normal pancreatic tissue (Fig. 1A). Based on different adjuvant chemotherapy regimen, 257 PDAC patients were divided into single gemcitabine group (GEM, 120 patients) and gemcitabine combined with albumin-bound paclitaxel group (GEM+ Nab-p, 137 patients). Among patients treated with GEM monotherapy, the disease control rate (DCR) between SOX8-high and SOX8-low group showed almost no difference (10% vs 7.6%) (Fig. 1B). However, among patients treated with GEM plus Nab-p combination therapies, the DCR of SOX8-low group (26.2%) was nearly twofold that of SOX8-high group (13.2%) (Fig. 1B), CT scans of PR, SD and PD patients were shown in Supplementary Fig. 1. The above data suggested that the expression of SOX8 maybe correlated with response to albumin-bound paclitaxel rather than gemcitabine.

Next, we analyzed the correlation between SOX8 expression and patients' survival in Gem or Gem+ albumin-bound paclitaxel patients, respectively. We found that patients with high SOX8 expression showed a shorter RFS survival than those with lower SOX8 expression in patients treated with GEM plus Nab-p (SOX8 high: 14.45 months vs SOX8 low: 19.09 months, p=0.02) (Fig. 1C). No correlation between SOX8 expression and RFS was found among patients treated with GEM alone (14.11 months vs 12.60 months, p=0.2) (Fig. 1C). Given the role of SOX8 in affecting albumin-bound paclitaxel sensitivity, we checked the expression difference of SOX8 in Nab-p treatment group based on response to the drug. We found SOX8 expression is low in those patients with significant response to albumin-bound paclitaxel (DCR group, p<0.001), from which we could infer SOX8 expression is potentially related to Nab-p treatment (Fig. 1D). Patient-derived xenograft (PDX) cell lines were set up from four patients based on response to albumin-bound paclitaxel (2 Nab-p sensitive patients: PDX0001 and PDX0037; 2 Nab-p insensitive patients: PDX0015 and PDX0049). We found expression of SOX8 was low in PDX0001 and PDX0037 while high in PDX0015 and PDX0049 (Fig. 1E) and the same trend appears in PDX tumor tissues (Supplementary Fig. 2). The primary tumor cells were treated with albumin-bound paclitaxel for 72 hours. CCK-8 demonstrated the cell viability of PDX0015 and PDX0049 was significantly higher than that of PDX0001 and PDX0037, which indicated the influence of SOX8 on Nab-p treatment. The results of flow cytometry showed that the ratios of G2 phase in PDX0001 (80.3%±4.9%) and PDX0037 (84.97%±5.3%) was significantly higher than PDX0015 (60.97%±2.9%) and PDX0049 (36.97%±4.7%) (Fig. 1G). The results inferred that Nab-p treatment induced cell cycle arrest at G2 phase while SOX8 may alleviate the effect. Taken together, our data indicated that SOX8 correlated with response to Nab-p treatment in PDAC. And we also found that the growth capacity of cells was positively correlated with the expression level of SOX8 (Supplementary Fig. 3).

### SOX8 reduced apoptosis caused by albumin-bound paclitaxel

**Figure 2:**
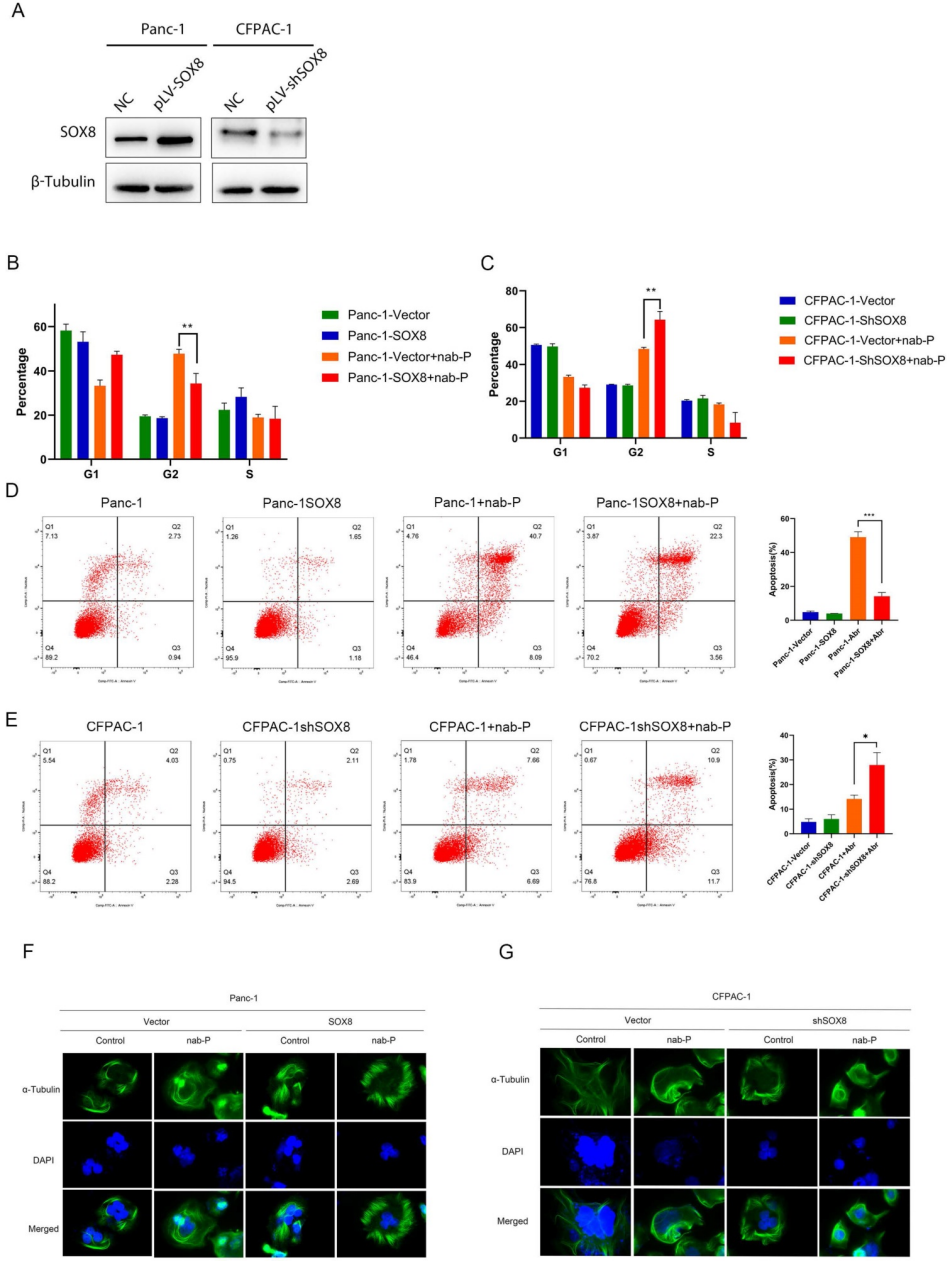
** SOX8 reduced apoptosis induced by nab-P. (A)** SOX8 expression in Panc-1 overexpressing SOX8 and SOX8 knock-down CFPAC-1 cells. **(B)** Apoptosis and **(C)** cell cycle and **(F)** microtubles of Panc-1 overexpressing SOX8 treated with nab-P. **(D)** Apoptosis and **(E)** cell cycle and **(G)** microtubles of SOX8 knock-down CFPAC-1 cells treated with nab-P.

According to the expression level of SOX8 in each pancreatic cancer cell line (Supplementary Fig. 4), we constructed stable SOX8 overexpressing Panc-1 cell line (Panc-1 SOX8) and Sox8 knockdown CFPAC-1 cell line (CFPAC-1 shSOX8) (Fig. 2A). The above cells were treated with Nab-p for 72h. Flow cytometry was used to examine cell cycle and apoptotic rate. G2 phase arrest was more significant in Panc-1 cells (47.80%±1.97%) compared with Panc-1 SOX8 cells (34.30%±4.56%, P<0.05) (Fig. 2B). CFPAC-1 shSOX8 cells (64.30%±4.56%) have increased G2 arrest than CFPAC-1 cells (48.47%±1.90%, P<0.05) when treated with Nab-P (Fig. 2C). Cell apoptosis was significantly reduced in Panc-1 SOX8 cells compared with Panc-1 cells (49.08%±3.21% vs 14.15%±2.29%, P<0.05) (Fig. 2D). Conversely, more apoptotic cells were exhibited in in CFPAC-1 shSOX8 cells than CFPAC-1 cells (44.31%±2.10% vs 22.48%±3.21, P<0.05) (Fig. 2E). Nab-p may bind with α-Tubulin to stabilize microtubules and inhibit the microtubles dynamics. Cells sensitive to Nab-p may show a compact pattern of α-Tubulin. With Nab-p treatment, Panc1 SOX8 cells showed a more -expression may diminish Nab-p treatment efficacy while knock down of SOX8 may increase sensitivities to Nab-p.

### SOX8 reduced the uptake of albumin-bound paclitaxel by reducing the expression of SPARC

**Figure 3:**
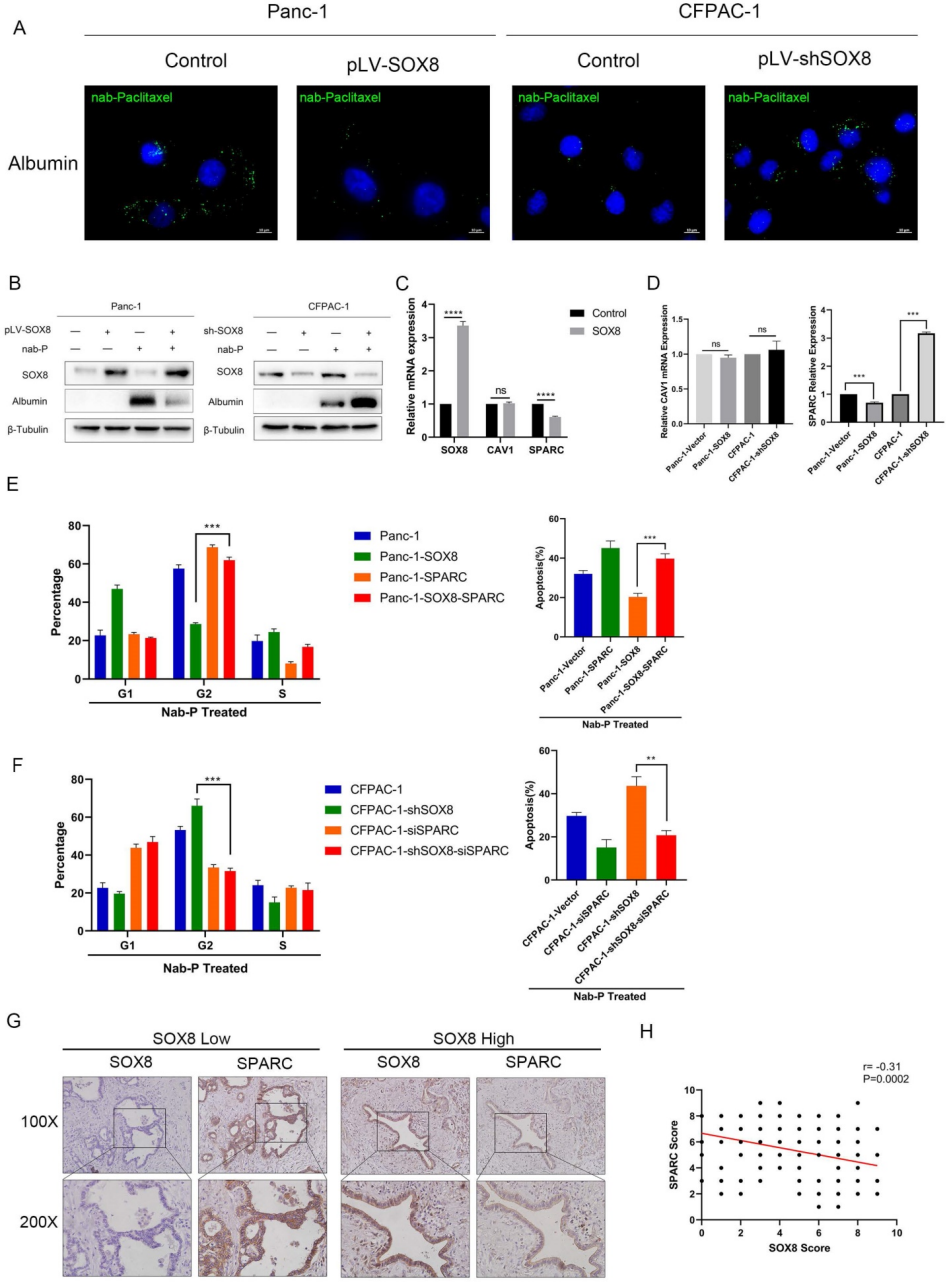
** SOX8 reduced the uptake of albumin-bound paclitaxel by reducing the expression of SPARC. (A)** Uptake of albumin-bound paclitaxel of SOX8 overexpression Panc-1 cells and SOX8 knock-down CFPAC-1 cells. **(B)** Western Blot analysis of albumin-bound paclitaxel taken into cells using anti-albumin antibody. **(C)** mRNA expression of SOX8/CAV1/SPARC in transcriptomic sequencing results. **(D)** mRNA expression 0f CAV1/SPARC in SOX8 overexpression Panc-1 cells and SOX8 knock-down CFPAC-1 cells. **(E)** Cell cycle and apoptosis of Panc-1-SOX8 and Panc-1 -SOX8-siSPARC. **(F)** Cell cycle and apoptosis of CFPAC-1-shSOX8 and CFPAC-1-shSOX8-SPARC. **(G)** Immunohistochemical co-localization of SOX8 and SPARC and **H.** Statistical Analysis,

To explore the mechanism by which SOX8 affect sensitivity to Nab-p treatment in PDAC, we tested whether SOX8 would affect albumin uptake in tumor cell. By immunofluorescence, we could readily detect albumin uptake when Panc1 and CFPAC-1 shSOX8 cells were pulsed with 0.5% (w/v) human albumin for 30 min in medium (Fig. 3A) but could not detect albumin in Panc1 SOX8 cells and CFPAC-1 tumor cells (Fig. 3A). To directly determine if SOX8 alters albumin uptake in tumor cell lines, we added Nab-p to control, SOX8 overexpression and SOX8 knockdown cells and performed immunoblotting with a human specific primary antibody to albumin. As shown in Fig. 3B, overexpression of SOX8 resulted in significant reductions in albumin uptake when cells were treated for 0.5 hours with Nab-p, vice-versa. Previous studies have shown that albumin enters the cell through caveolin 12, which was consisted of CAV-1 and SPARC. Our transcriptomic sequencing results showed that SPARC, rather than CAV1 was significantly reduced in SOX8 overexpression group (Fig. 3C). Further, we detected the expression of SPARC and CAV1 by q-PCR and found SPARC expression was altered followed by SOX8 change (Fig. 3D) and changes in protein levels of SPARC and CAV1 were consistent with mRNA levels (Supplementary Fig. 5). Next we knocked down SPARC in CFPAC-1 and CFPAC-1 shSOX8 cells (CFPAC-1-shSOX8-siSPARC) and upregulated SPARC in Panc-1 and Panc-1-SOX8 (Panc-1-SOX8-SPARC) cells. Then we treated the above cells with Nab-p and examine the G2/M phase arrest and apoptosis rate. The results showed that SPARC overexpression may restore sensitivity to Nab-p treatment-induced G2/M arrest and apoptosis in Panc-1-SOX8 cells (Fig. 3E). Conversely, SPARC knock down would diminish the cell cycle arrest and apoptosis in Nab-p treated CFPAC-1-shSOX8 cells (Fig. 3F). The dual staining of SOX8 and SPARC by IHC (Fig. 3G) and statistical analysis (Fig. 3H) proved that the expression of SPARC was negatively correlated with SOX8 in PDAC patients.

### SOX8 up-regulates the Expression of EZH2

**Figure 4:**
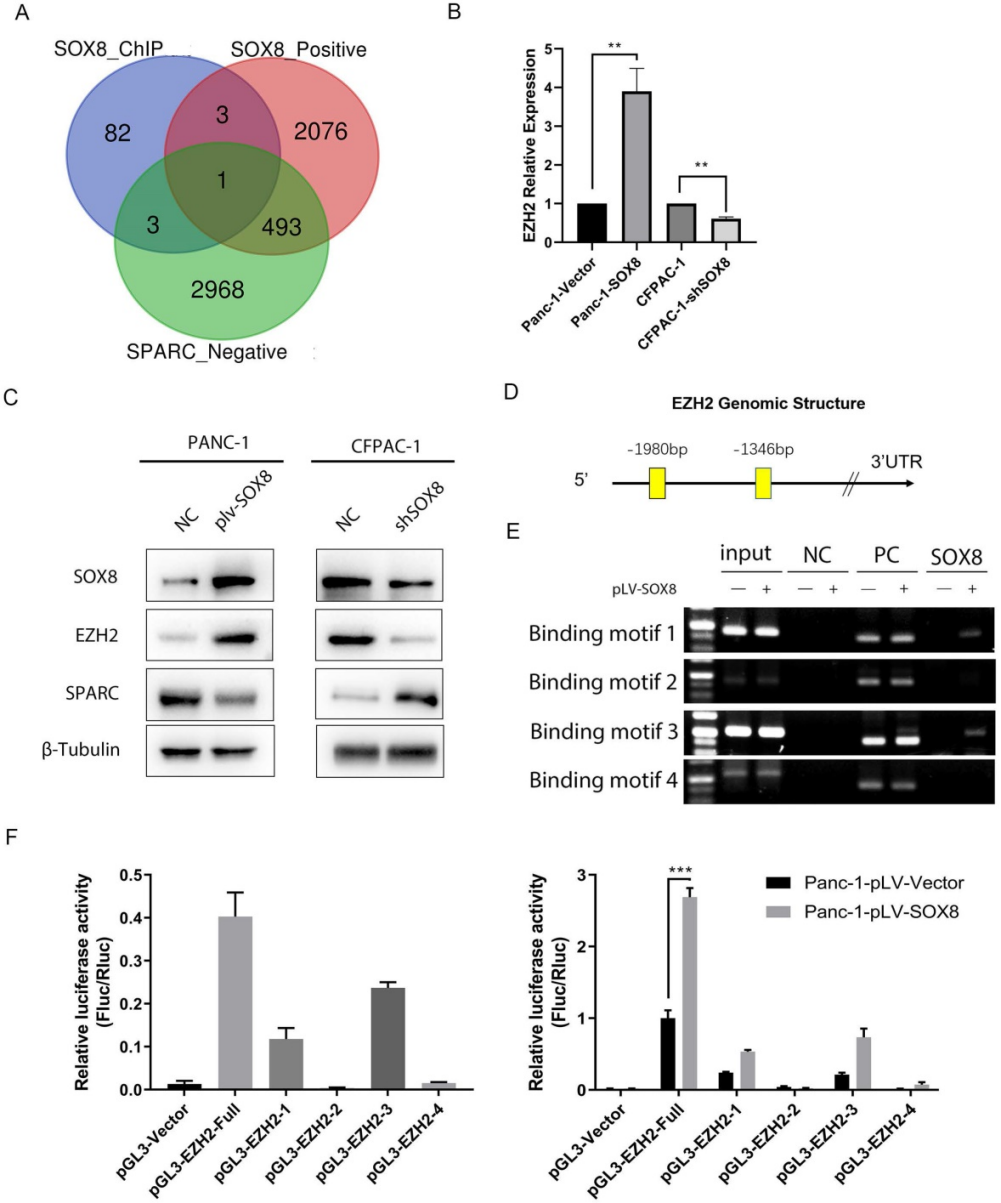
** SOX8 up-regulate the expression of EZH2. (A)** Venn diagram of sequencing data **(B)** mRNA expression of EZH2 in Panc-1-SOX8 and CFPAC-1-sh SXO8 cells and **(C)** protein level. **(D)** Potential binding sites region of SOX8 in EZH2 promoter. **(E)** ChIP results in Panc-1-SOX8 cells (NC: Normal Mouse IgG was used in the Immunoprecipitation (IP) of Crosslinked Protein/DNA, PC: anti-RNA Polymerase was used in the IP of Crosslinked Protein/DNA, and GAPDH Positive Control primer was added into PCR reaction, SOX8: SOX8 antibody was used in the IP of Crosslinked Protein/DNA, and EZH2 primer was added into PCR reaction) **(F)** Luciferase analysis in 293T cells and Panc-1-SOX8 cells.

Given the correlation between SOX8 and SPARC, we sought to find to mechanism of SOX8 to regulate SPARC. As a transcription factor, SOX8 could not directly regulate SPARC due to the absence of SOX8 binding motif in promoter region of SPARC. We performed Chromatin immunoprecipitation (ChIP) sequencing and transcriptome sequencing. By searching the intersection between genes transcriptionally regulated by SOX8, genes positively correlated with SOX8 and genes negatively correlated with SPARC, we found enhancer of zeste 2 polycomb repressive complex 2 subunit (EZH2) was the only candidate meet all the criteria (Fig. 4A). We performed q-PCR and Western Blot to detect the expression of EZH2 in Panc-1/Panc-1-SOX8 and CFPAC-1/CFPAC-1-shSOX8. The results showed expression of EZH2 up-regulated in Panc-1-SOX8 cells both in mRNA level (Fig. 4B) and protein level (Fig. 4C). Conversely SOX8 knockdown downregulated EZH2 in CFPAC-1. We surveyed the promoter region of human EZH2 gene and identified four potential binding sites of SOX8 between -1346 to -1980 (Fig. 4D).

To investigate whether SOX8 directly binds to EZH2 promoter, chromatin immunoprecipitation assay was performed in Panc-1 cells. In chromatin fractions pulled down by an anti-SOX8 antibody, only the binding site of EZH2 promoter located at -1891 to -1905 and -1457 to -1474 was detected (Fig. 4E). The fragment immunoprecipitated by anti-SOX8 antibody significantly increased (Fig. 4F left; P < 0.01), suggesting the binding of SOX8 to EZH2 promoter.

To determine whether the binding of SOX8 activates EZH2 promoter, we constructed a full-length EZH2 luciferase promoter vector (containing binding sites, -1891 to -1905 and -1457 to -1474) and co-transfected this reporter construct with or without SOX8 cDNA into Panc1 cells. Luciferase analysis showed that SOX8 overexpression (pLV-SOX8) significantly increased EZH2 promoter activity in Panc-1 cells (2.9-fold, P < 0.05; Fig. 4F right). To determine whether the SOX8 binding site is required for SOX8 to transactivate EZH2 promoter, this SOX8 binding site was mutated from ACGTG to AAAAA. As shown in Fig. 4F, the mutation of SOX8 binding sites almost abolished the transactivation of EZH2 promoter by SOX8. From above data we showed that SOX8 up-regulated EZH2 by transcriptional regulation.

### EZH2 reduced the expression of SPARC

**Figure 5:**
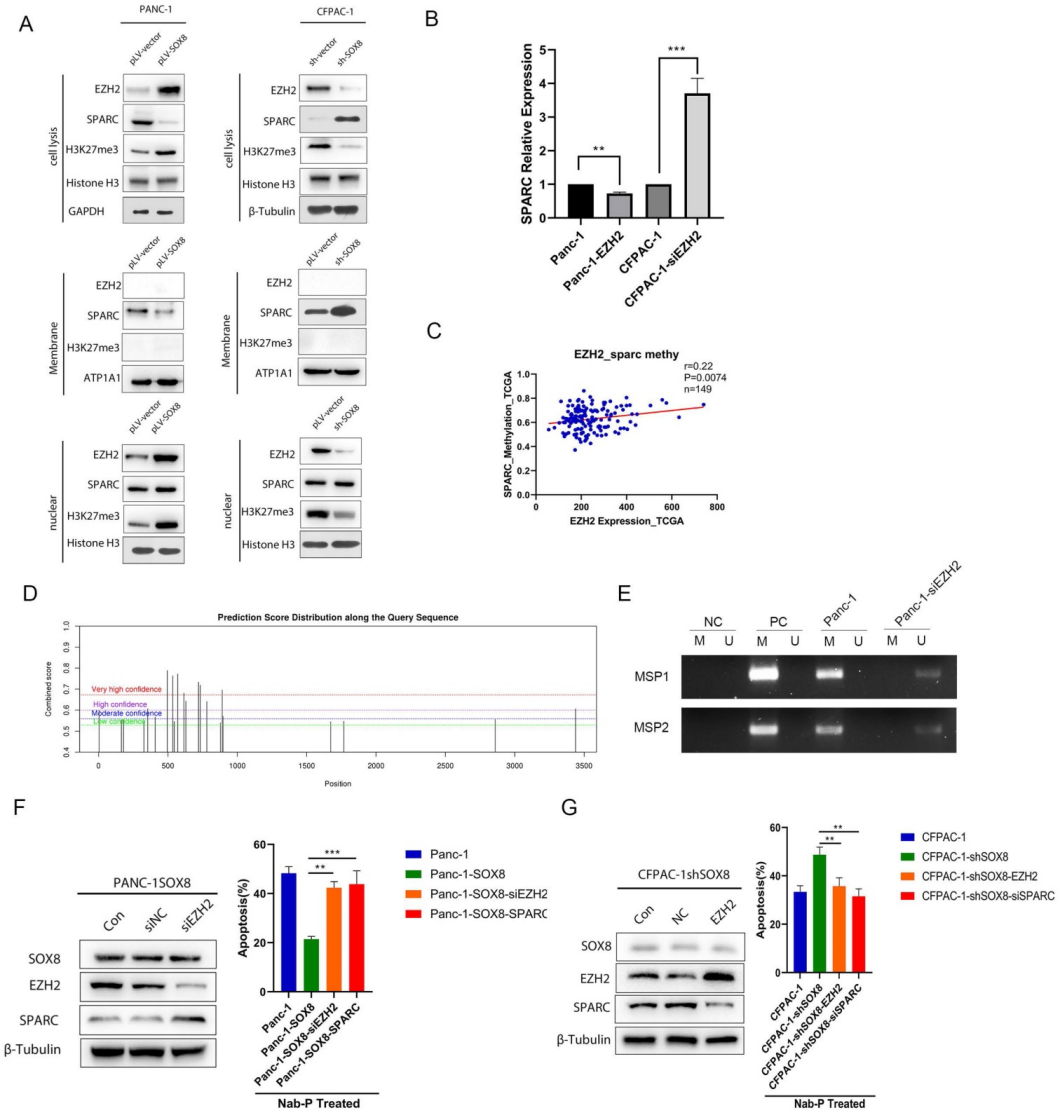
** EZH2 reduces the expression of SPARC. (A)** Expression of EZH2/SPARC/H3K27me3 on the membrane of PDAC cells. **(B)** mRNA expression of SPARC in Panc-1-EZH2 and CFPAC-1-siEZH2 cells.** (C)** Correlation of EZH2 expression and SPARC methylation in TCGA database. **(D)** Potential m6A site of SPARC. **(E)** Methylation specific PCR results of MSP1 and MSP2. **(F)** Protein expression of SPARC in Panc-1-SOX8-siEZH2 and apoptosis rate. **(G)** Protein expression of SPARC in CFPAC-1-shSOX8-EZH2 and apoptosis rate.

To further determine whether SOX8 might regulate SPARC through EZH2, we isolated the nucleus and membrane protein from stable lines with altered SOX8. Western Blot was used to detect the expression of EZH2 and SPARC in the nucleus and cell membrane respectively. We found EZH2 and H3K27 methyltransferase were upregulated in the nucleus while SPARC was down-regulated on the membrane following SOX8 up-regulation (Fig. 5A). On the other hand, nuclear EZH2 and H3K27 methyltransferase were decreased while membranous SPARC was increased when SOX8 was knockdown (Fig. 5A). The alteration of EZH2 would lead SPARC expression to change in an opposite direction at transcriptional level (Fig. 5B). We analyzed the possible m6A site in the mRNA of SPARC by SRAMP. The results showed there are 22 possible sites. We selected the top 2 scored sites and named them as MSP1 and MSP2 respectively. Methylation specific PCR was adopted and results showed MSP1 and MSP2 was hypermethylated in Panc-1 cells (Fig. 5E). In the bottle-neck experiment, immuno-blot result showed that knocking down of EZH2 in SOX8 expressing Panc-1 may lead to regain of SPARC expression (Fig. 5F). However, up-regulation of EZH2 in CFPAC-1 shSOX8 cell may decrease the expression of SPARC (Fig. 5G). When treated with Nab-p, either EZH2 knock down (42.37%±2.51%, P<0.05) or SPARC up-regulation (43.83%±5.45%, P<0.05) would cause more robust apoptosis in SOX8 expressing Panc-1 cell (21.40%±1.15%). On the contrary, the apoptosis rate in CFPAC-1 shSOX8 cells (48.73%±3.43%) would reduce if EZH2 was up regulated (35.64%±2.51%, P<0.05) or SPARC was knocked down (31.53%± 3.10%, P<0.05).

### Inhibition of EZH2 increased the sensitivity of pancreatic cancer to albumin-bound paclitaxel

**Figure 6:**
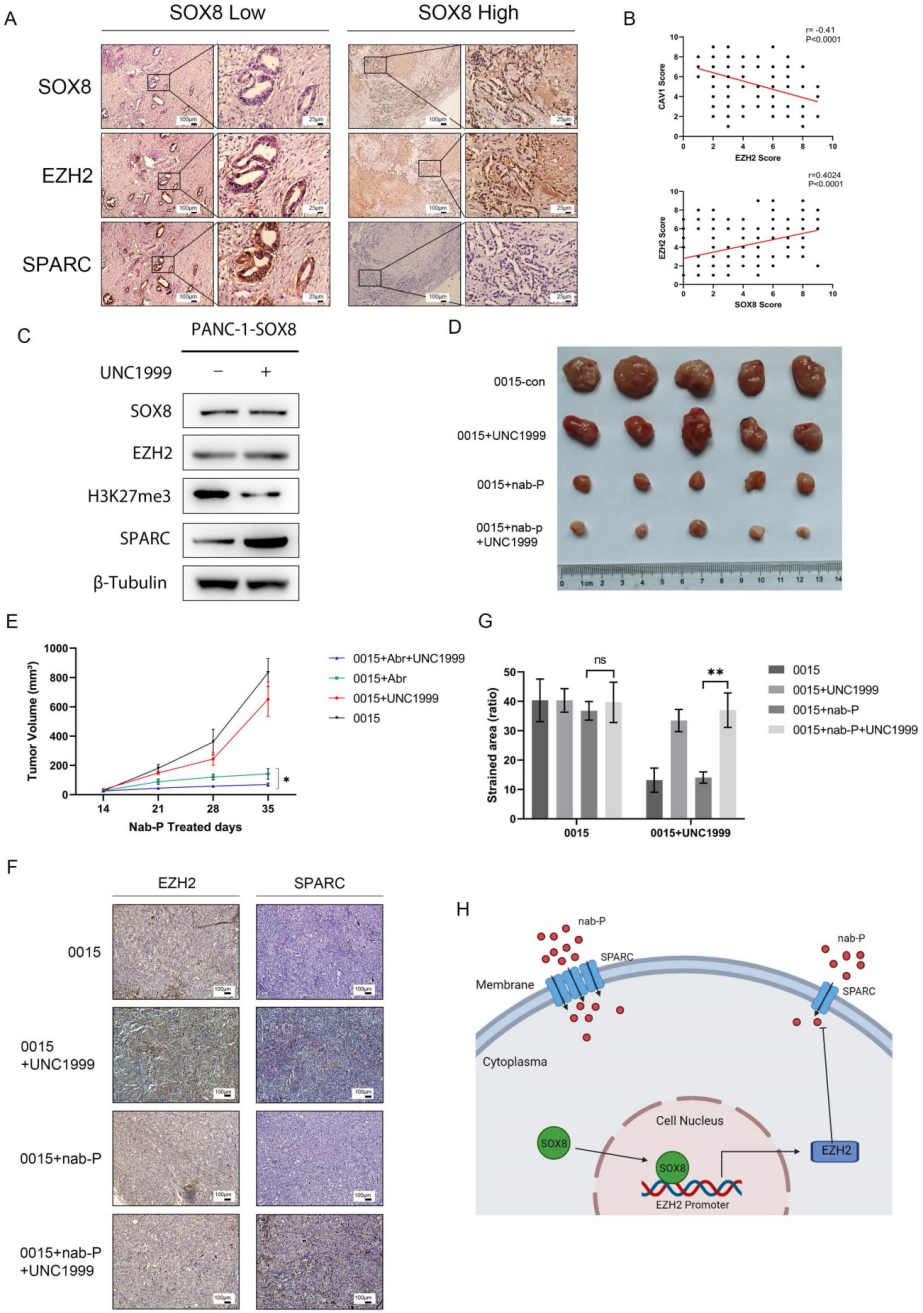
** Inhibition of EZH2 increased the sensitivity of pancreatic cancer to albumin-bound paclitaxel. (A)** Dual staining of SOX8, EZH2 and SPARC and (B) Statistical Analysis. **(C)** SPARC expression in Panc-1-SOX8 cells treated with UNC1999. **(D)** Picture of tumors and **(E)** tumor volume. **(F)** IHC straining of EZH2/SPARC of primary tumor and **(G)** Statistical Analysis. **(H)** Working model.

Co-localization staining was performed to verify the correlation of SOX8, EZH2 and SPARC (r=0.40, P<0.0001, Fig. 6A). Statistical analysis showed a positive correlation between SOX8 and EZH2 as well as a negative correlation between EZH2 and SPARC (r=-0.41, P<0.0001, Fig. 6B). UNC1999 was a bioavailable inhibitor that has high *in vitro* potency for wild type and mutant EZH2, which was a closely related to H3K27 methyltransferase. Expression of SPARC was increased in SOX8 expressing cells treated with UNC1999 followed by H3K27me3 inhibition (Fig. 6C), and the status of H3K27me3 was also affected by the change of EZH2 expression (Supplementary Fig. 6). Subcutaneous tumor formation was performed in nude mice with PDX0015 line, which showed a high level of SOX8 expression. Then these mice are divided into four groups, treated with normal saline, UNC1999, albumin-bound paclitaxel and albumin-bound paclitaxel combined with UNC1999. *In vivo* study showed that tumor shrink was most significant in albumin-bound paclitaxel combined with UNC1999 group compared with the other three groups (Fig. 6D & Fig. 6E). IHC staining of primary tumor displayed that although EZH2 expression level was not change while SPARC expression was increased in UNC1999 group and albumin-bound paclitaxel combined with UNC1999 group (Fig. 6F & Fig. 6G), which indicated that inhibition of EZH2 methylation may reinforce the effect of albumin-bound paclitaxel by increase SPARC expression. The working model was showed in Fig. 6H.

## Discussion

A wide variety of findings have shown that SOX genes act as oncogenes or tumor suppressors are involved in tumor formation and progression 13. SOX8 is one of the members of the SOXE family, which plays crucial roles in the peripheral and central nervous systems as regulators of stemness, survival, homeostasis and glial differentiation 14. In the present study, we demonstrated that SOX8 is upregulated in PDAC and our results uncover a novel mechanism mediated by the SOX8/EZH2/SPARC axis in PDAC that lead to albumin-bound paclitaxel primary chemoresistance, which lead to a poor prognosis for PDAC patients.

### SOX8 is a transcription factor containing a DNA binding region named HMG box 15

It has been reported to be overexpressed and acts as an oncogene in liver cancer 16 and breast cancer 9. Notably, SOX8 plays an important role in chemotherapy resistance of gestational trophoblastoma, and it participates in multiple drug-resistant pathways 17. In the present study, we found that PDAC patients with high SOX8 expression exhibited poor response to albumin-bound paclitaxel chemotherapy through cohort study and validation of our hospital PDAC cancer tissue. After knockdown or overexpression of SOX8 in different cell lines, we identified SOX8 as a functional oncogene that is involved in the inducement of primary resistance to albumin-bound paclitaxel in PDAC cells.

Currently, albumin-bound paclitaxel is the first line option for chemotherapy for PDAC patients 18. Secreted protein acidic and rich in cysteine (SPARC), also known as Osteonectin or basement membrane protein 40 (BM-40), is a glycoprotein which facilitates the accumulation of albumin-bound paclitaxel in tumors 19. In the study, we found that expression of SPARC and SOX8 was negatively correlated. Regain of SPARC in SOX8 expressing cells may recover the sensitivity of PDAC cells to albumin-bound paclitaxel. There have been many reports on the regulation of SPARC. The abnormal methylation of the SPARC gene CpG islands is found in 28% of resected PanIN tissue 20. In our study two hypermethylation wave peak regions were found in SPARC gene transcriptional regulation region. ChIPs, as well as transcriptome sequencing data showed EZH2 was the bridge connected SOX8 and SPARC methylation. *In vitro* study revealed that SOX8 increases the methylation of the SPARC promoter region by directly regulating EZH2 at transcriptional level, which led to decrease of membranous SPARC expression and attenuated the effect of albumin-bound paclitaxel. Further, the SOX8/EZH2/SPARC axis was validated in TCGA database and PDAC tissue from our center. Moreover, *in vivo* study illustrated the inhibition of methylation function of EZH2 may recover SPARC expression in SOX8 expressing cells and reverse the albumin-bound paclitaxel primary chemoresistance.

In conclusion, our research uncovered SOX8/EZH2/SPARC signaling drives albumin-bound paclitaxel resistance in PDAC. Therefore, SOX8 may be regarded as a biomarker to predict the response to albumin-bound paclitaxel. For SOX8 overexpression patients, EZH2 methylation inhibitor turns to be an optimal combination of albumin-bound paclitaxel as a novel strategy for PDAC patients.

## Supplementary Material

Supplementary figures and tables.

## Abbreviations

PDAC: Pancreatic ductal adenocarcinoma
Nab-p: albumin-bound paclitaxel
SPARC: secreted protein acidic and rich in cysteine
SOX: Sex-determining region Y-boxes
EZH2: enhancer of zeste homolog 2
GEM: gemcitabine
PDX: Patient-derived xenograft
