# Supplementary material for: SOX8 Affects Tumoral SPARC Expression by Regulating EZH2 to Attenuate Effectiveness of albumin-bound paclitaxel in PDAC
Source: Int J Biol Sci. 2022 Jan 1;18(3):911–22. doi: 10.7150/ijbs.64752 (PMC8771850; doi:10.7150/ijbs.64752)
Supplement: Supplementary file 1 — Supplementary figures and tables. [file ijbsv18p0911s1.pdf]

Supplementary Fig 1

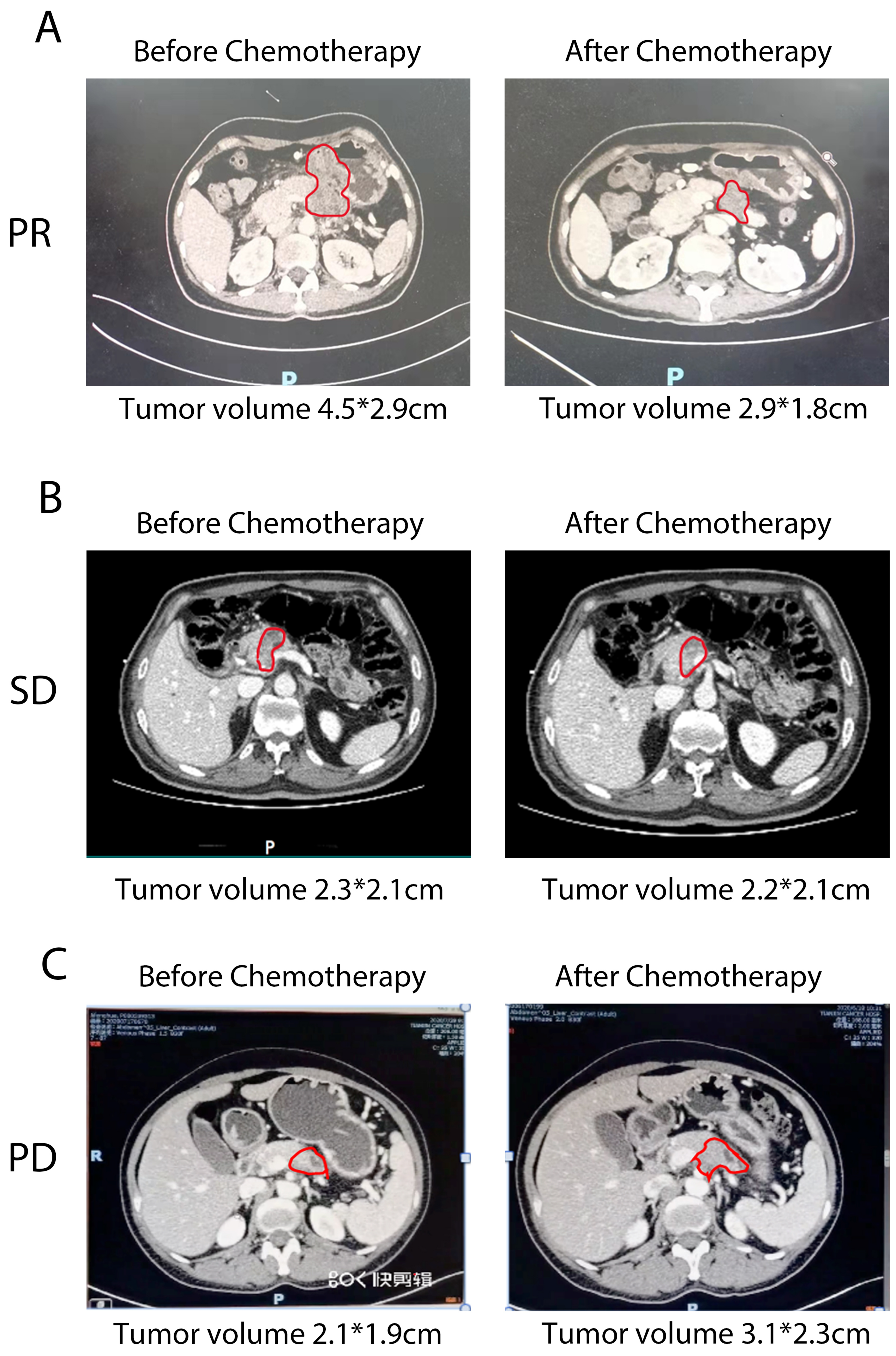

Supplementary Fig 2

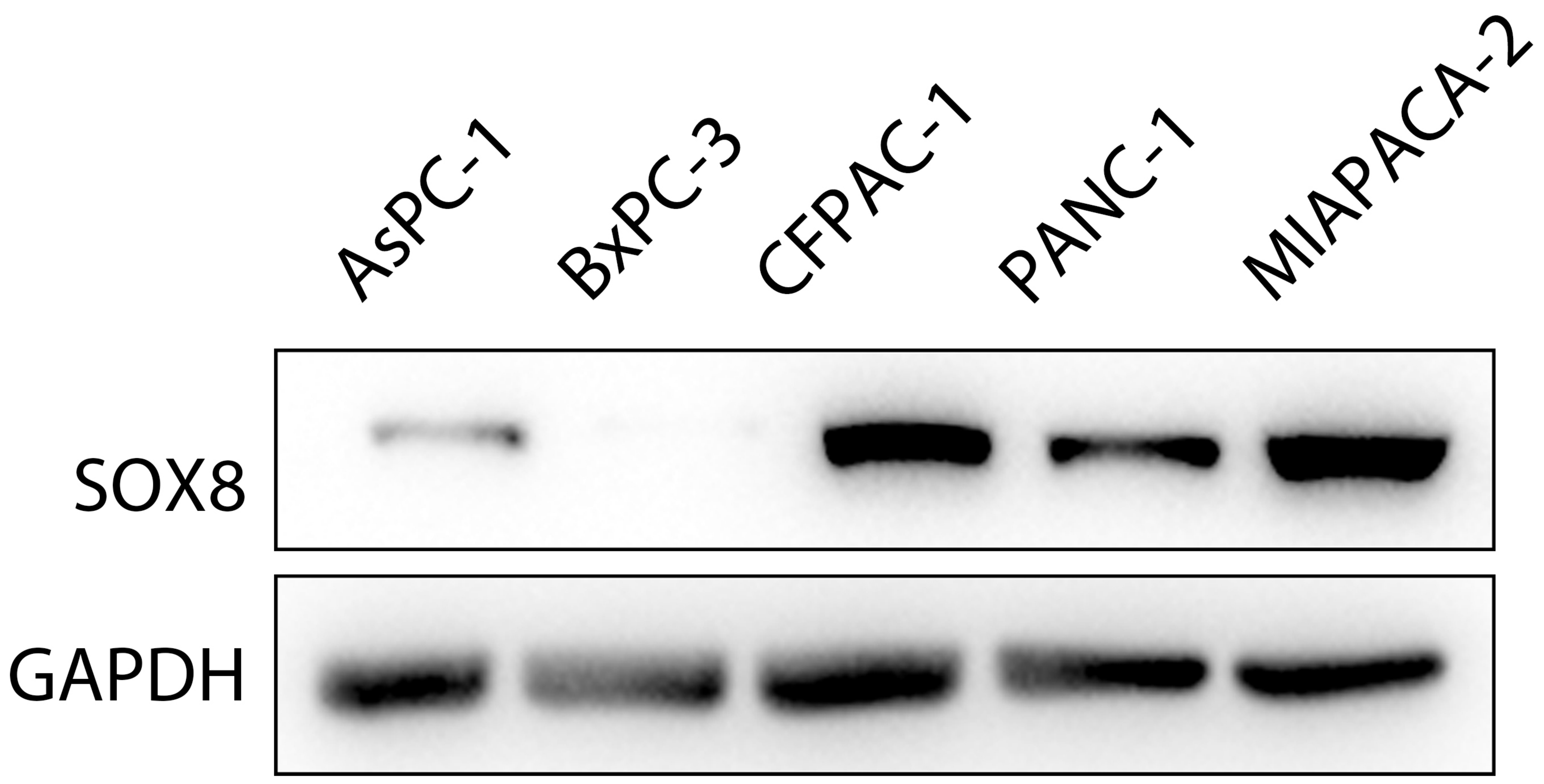

Supplementary Fig 3

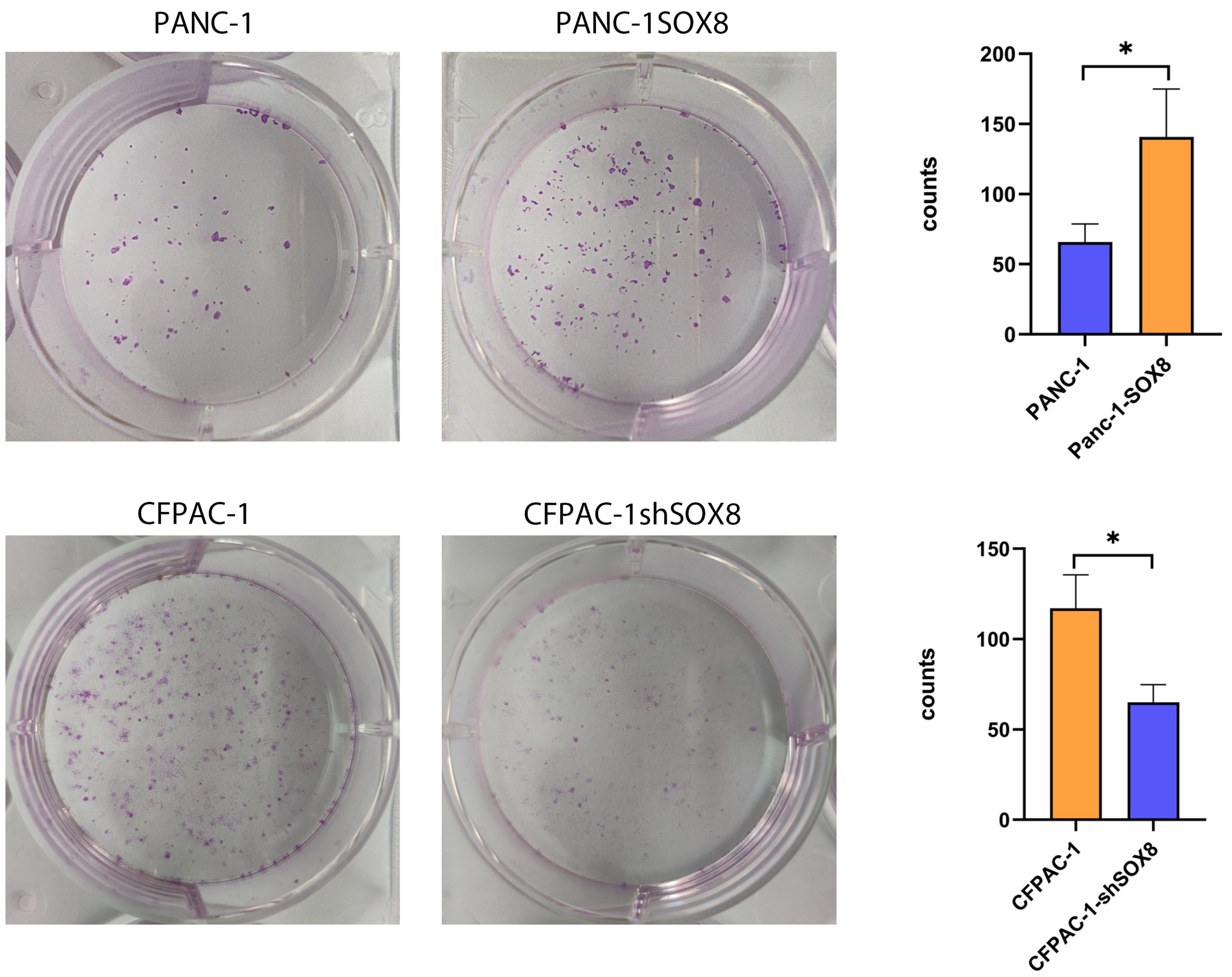

Supplementary Fig 4

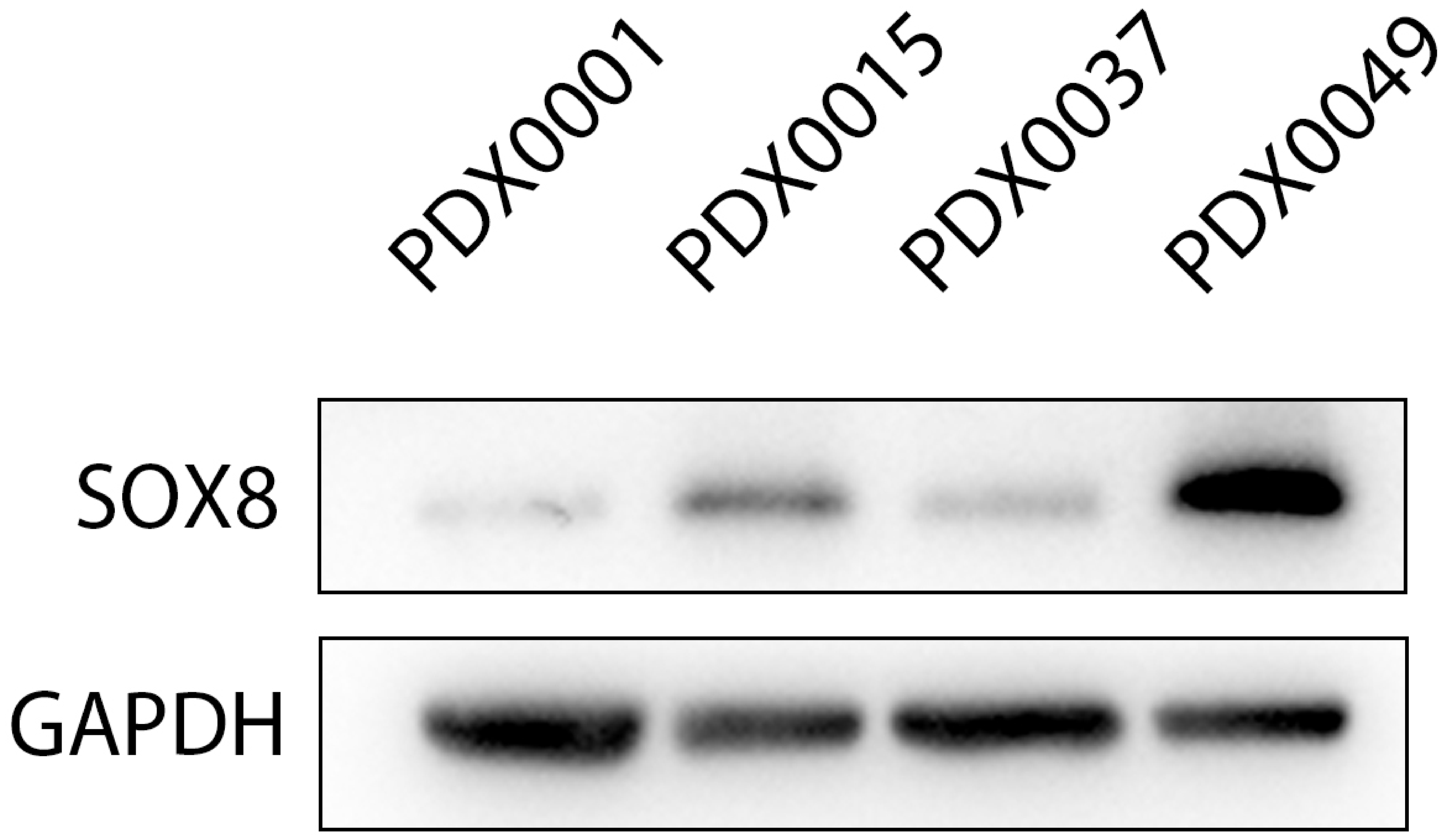

Supplementary Fig 5

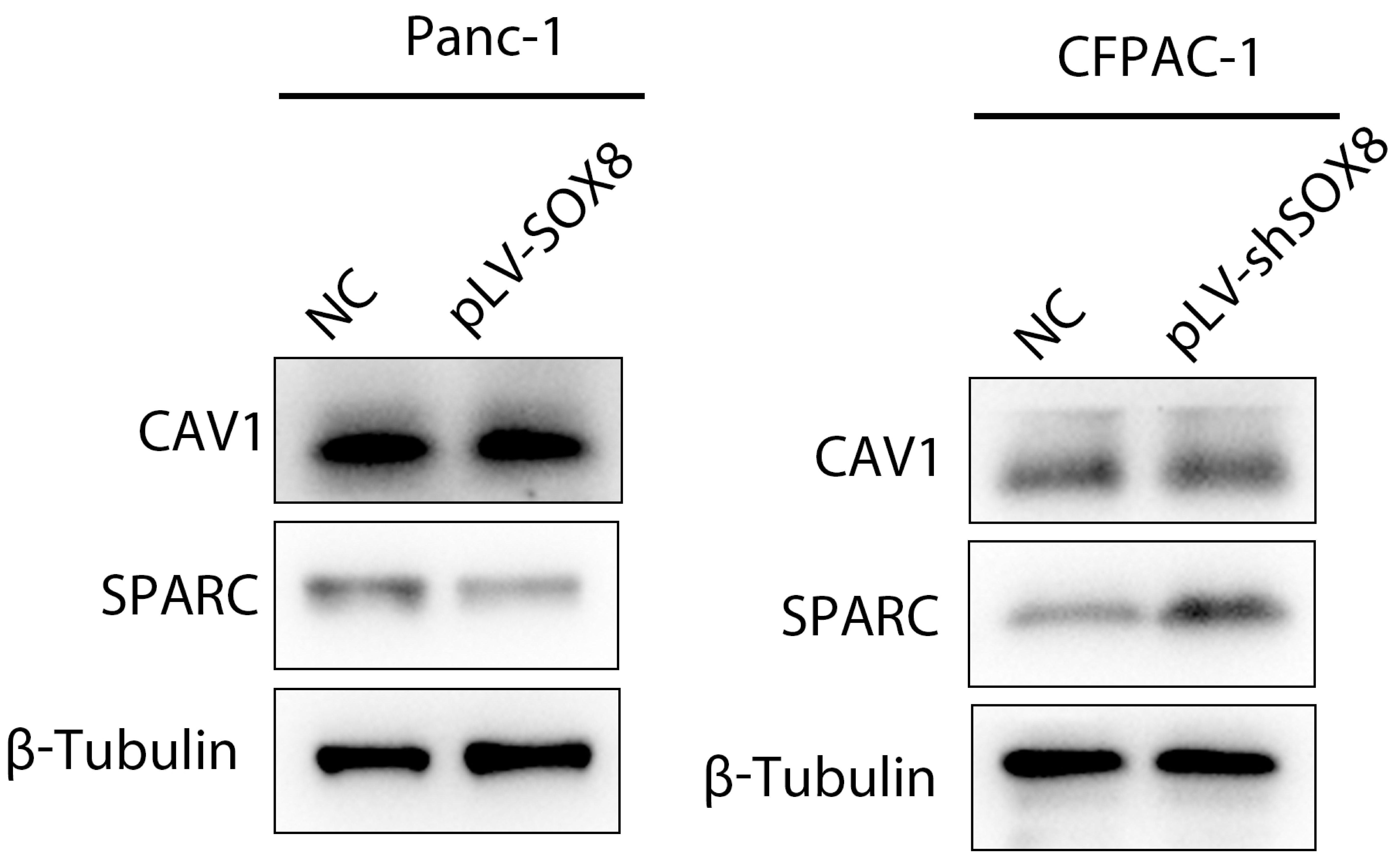

Supplementary Fig 6

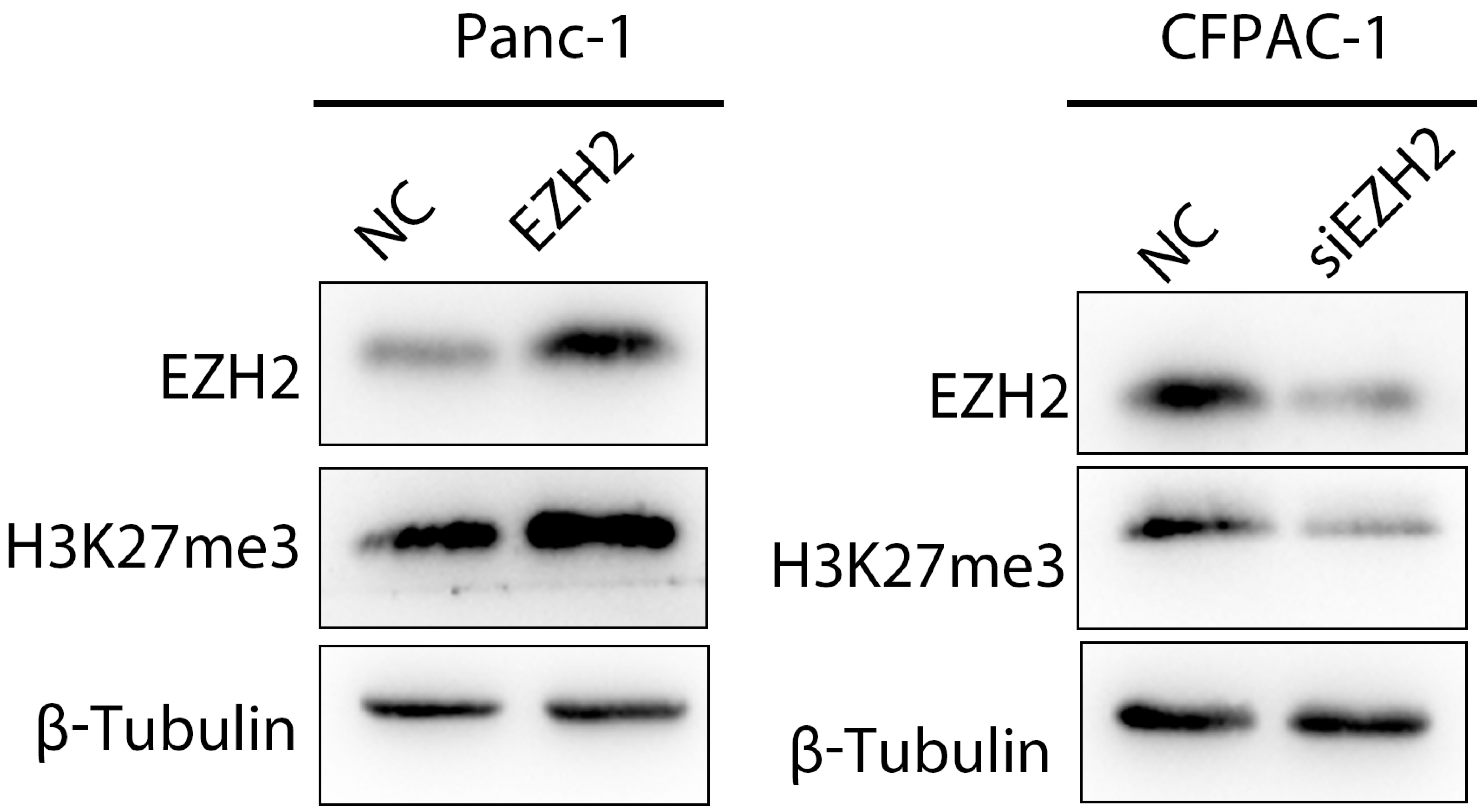

Table S1 Antibodies

	Manufactor	Catalog no
GAPDH	Ray antibody biotech， China	RM2002
β-Tubulin	Ray antibody biotech， China	RM2003
β-actin	Ray antibody biotech， China	RM2001
SOX8	Abcam, USA	ab104245
Albumin	Abcam, USA	ab207327
EZH2	CST, USA	5246
SPARC	Abcam, USA	ab207327
Histone H3	Ray antibody biotech， China	RM2005
ATP1A	Proteintech, China	14418-1-AP

Table S2 Primers

Gene	Forward primer	Reverse primer
SOX8	CAGTACCCCTGCTTCCACTC	AGACTTCAGCAGGCACTTGG
EZH2	AGGAGTTTGCTGCTGCTCTC	CCGAGAATTTGCTTCAGAGG
SPARC	CAAAACGCAGCTCCCCAATC	GCCCATTGCAATTGAGCACA
β-actin	CAGAGCAAGAGAGGCATCC	CTGGGGTGTTGAAGGTCTC

Table S3 small interfering RNA

Gene	Sequence
EZH2	AACAAGACCUUCGACUCUUCC
SPARC	AGGAGGTGCAGACTAGCTGAA
siScramble	GCUCACAGCUCAAUCCUAAUC
